# Inflammatory markers C-reactive protein and PLR in relation to HCC characteristics

**DOI:** 10.15761/JTS.1000260

**Published:** 2018-06-22

**Authors:** Aslı Suner, Brian I Carr, Hikmet Akkiz, Oguz Uskudar, Sedef Kuran, Yaman Tokat, Salih Tokmak, Tugsan Ballı, Abdulalh Ulku, Tolga AkCam, Anıl Delik, Burcu Arslan, Figen Doran, Kendal YalCın, Nazım Ekinci, Sezai Yilmaz, Ayşegul Ozakyol, Mehmet Yücesoy, Halil Ibrahim BahCeci, Kamil Yalcın Polat, Halis Şimsek, Necati Ormeci, Abdulalh Sonsuz, Mehmet Demir, Murat KılıC, Ahmet Uygun, Ali Demir, Engin Altıntas, Gokhan Karakulah, Tuncer Temel, Ahmet Bektas

**Affiliations:** 1 Ege University, Faculty of Medicine, Department of Biostatistics and Medical Informatics, İzmir, Turkey; 2 Liver Transplant Inst, Inonu University, Malatya, Turkey; 3 Cukurova University, Gastroenterology Department, Adana, Turkey; 4 Cukurova University, Rektorlugu, 01330 Sarıcam/Adana, Turkey; 5 Dicle University, 21280 Sur/Diyarbakir, Turkey; 6 Inonu University Malatya, 44210 Battalgazi/Malatya, Turkey; 7 Eskisehir Osmangazi University, Meselik Yerleskesi, 26040 Odunpazarı/Eskisehir, Turkey; 8 Erciyes University, Talas Blv., 38030 Melikgazi/Kayseri, Turkey; 9 Fırat University, 23119 Elazıg Merkez/Elazıg, Turkey; 10 Istanbul Memorial Hospital, Turkey; 11 Hacettepe University, Ankara, Turkey; 12 Ankara University, 06560 Yenimahalle/Ankara, Turkey; 13 Istanbul Cerrahpaşa University, Turkey; 14 Hatay Mustafa Kemal University, Turkey; 15 Izmir Kent hospital, Turkey; 16 Haydarpaşa Sultan Abdülhamid Egitim Araştırma Hospital, Turkey; 17 Konya Necmettin Erbakan University, Turkey; 18 Mersin University, Yenisehir/Mersin, Turkey; 19 Izmir International Biomedicine and Genome Institute, Dokuz Eylül University, Izmir, Turkey; 20 Eskişehir Gazi Osman Paşa University, Turkey; 21 Mayıs University, Turkey

**Keywords:** C-reactive protein, PLR, NLR, HCC, aggressiveness

## Abstract

**Introduction:**

Several markers of systemic inflammation, including blood C-reactive protein, platelet lymphocyte ratio (PLR) and neutrophil lymphocyte ratio (NLR) have been identified as independent prognosticators for hepatocellular carcinoma (HCC).

**Methods:**

To attempt to understand the significance of these markers, they were examined in relation to 4 tumour parameters, namely maximum tumour diameter (MTD), tumour multifocality, portal vein thrombosis (PVT) and blood alpha-fetoprotein (AFP) levels.

**Results:**

Using linear and logistic regression models, we found that C-reactive protein and PLR on single variables, were statistically significantly related to the tumour parameters. In a logistic regression final model, CRP was significantly related to MTD, AFP and PVT, and the Glasgow Index significantly related to MTD and AFP. Results of the area under the receiver operating characteristic curves (ROC), showed that the areas for PLR and CRP were statistically significant for high versus low MTD and for presence versus absence of PVT. CRP alone was significant for high versus low AFP.

**Conclusions:**

These analyses suggest that the prognostic usefulness of the inflammatory markers PLR and CRP (but not NLR) may be due to their reflection of parameter values for tumour growth and invasiveness.

## Introduction

C-reactive protein (CRP) is a recognized part of the acute phase response and is associated with various inflammatory diseases [1]. It is also considered to be a marker both of inflammation as well as of cancer [2,3]. Although it is secreted in the presence of HCC, it is not considered to be a diagnostic marker, but it has nevertheless been reported to have significant prognostic value [4–7]. The Glasgow inflammation score consisting of CRP and albumin, and has been shown to be an independent prognosticator for several cancer types, including HCC [8–17]. Furthermore, there is evidence that CRP is produced not just by hepatocytes, but also by HCC cells [18,19]. More recently, several other indices of inflammation, in particular the neutrophil-to-lymphocyte ratio and the platelet-to-lymphocyte ratio have been also suggested to be useful HCC prognosticators [20–29]. In this paper, we compare in a large Turkish HCC cohort, the neutrophil-to-lymphocyte ratio, the platelet-to-lymphocyte ratio and the C-reactive protein values (part of the Glasgow Index) and examine the relationship of all 3 indices to parameters of HCC tumour aggressiveness, in an attempt to explain the prognostic usefulness of these inflammatory indices.

## Methods

### Patient data

In this study, we analysed a database of 424 patients prospectively-accrued HCC patients who had full baseline tumour parameter data, including CT scan information on HCC size, number of tumour nodules and presence or absence of PVT, plasma AFP levels, complete blood count and routine blood liver function tests. Diagnosis was made either through tumour biopsy or according to international guidelines. Database management conformed to legislation on privacy and this study conforms to the ethical guidelines of the Declaration of Helsinki and approval for this retrospective study on de-identified HCC patients was obtained by the Institutional Review Board of each participating institution [30].

### Statistical analyses

The continuous variables including, maximum tumour diameter (MTD), alpha-fetoprotein (AFP) and CRP were divided into two groups with different cut-off values as less than 3 and ≥3, less than 20 and ≥20, and ≤10 and more than 10, respectively. Descriptive statistics for continuous variables, such as the neutrophils-to-lymphocyte ratio (NLR) and the platelet-to-lymphocyte ratio (PLR) for MTD, AFP, multifocality and portal vein thrombosis (PVT) groups were calculated with mean, standard deviation, median, interquartile range, minimum and maximum values. Shapiro-Wilk test was used to check the normality assumption of the continuous variables. In the cases of non-normally distributed data, the Wilcoxon rank-sum (Mann-Whitney U) test was performed to determine whether the difference between the two groups was statistically significant. A linear regression model was constructed to evaluate the associations between PLR on single variables. The univariate logistic regression method was utilized to assess the factors associated with CRP (≤10/>10) and Glasgow index (<2/=2) in single variables, and then multiple logistic regression method was performed. All final multiple logistic regression models were executed with the backward stepwise method. The ability of PLR, NLR and CRP values to predict MTD, AFP, Multifocality and PVT groups in HCC patients were examined by receiver operating characteristic (ROC) curve and their respective areas under the curve. A p-value of less than 0.05 was considered as statistically significant. All statistical analyses were performed using IBM SPSS version 21.0 (Chicago, IL, USA).

## Results

### Descriptive statistics of PLR, NLR and CRP in relation to tumour characteristics

The focus of this study was 424 HCC patients with full data including CRP levels. Mean PLR, NLR and CRP were 0.16±0.13, 4.53±4.66 and 18.44±33.24, respectively (data not shown) (Table 1). summarizes the descriptive statistics and comparisons of the NLR ratio, PLR ratio and CRP levels for each of the 4 tumour characteristics of MTD, AFP, multifocality and PVT patient groups. The PLR and the CRP in the MTD≥3cm group were statistically significantly higher than the MTD<3cm group (p = 0.002 and p = 0.001, respectively). The CRP level, but not the NRL and PLR ratios in the AFP≥20 IU/ml group were found to be significantly increased compared to the AFP<20 IU/ml group (p<0.001). We also found that the PLR ratio and the CRP levels were significantly greater in the patients with presence of PVT compared with the PVT absent group (p = 0.034 and p<0.001, respectively). Our statistical analysis indicates that NLR levels were not significantly different among MTD, AFP, multifocality or PVT groups (p>0.05). However, when multifocality compared to unifocality, neither NLR nor CRP were significantly altered between the 2 groups, and the PLR was actually lower in the multifocal patients than the unifocal ones (p = 0.041).

### Regression models on single variables and final models for tumour parameters

Linear regression models were then constructed for the association between the PLR ratio and the four tumour parameters, separately (Table 2A). Based on our regression models (Table 2A), MTD was the only statistically significant parameter (p = 0.004). A univariate logistic regression models of the CRP groups (CRP≤10 and >10) provided distinct p-values for each of the four tumour parameters, and MTD, AFP, and PVT were found to be statistically significant (Table 2B; p<0.05). In the final model of the CRP groups (Table 2D), significant associations were also detected between CRP groups and MTD, AFP and PVT (p<0.05). Similar results were obtained for univariate logistic regression models of Glasgow index, which is a composite of serum CRP plus serum albumin (Table 3C and 3E). All of the four tumour parameters were found to be statistically significant on single variable models for the Glasgow index (Table 2C), however only two of them (MTD and AFP) were statistically significant in the final model (Table 2E; p<0.05).

### Final models of logistic regression analysis for tumour characteristics

Final models of logistic regression analysis for independent variables the PLR, NLR ratios and CRP/Glasgow index were then built to evaluate their associations with each of the four tumour parameters (Table 3). This analysis revealed that the PLR and CRP/Glasgow index were significantly associated with MTD (Table 3A; p<0.05). In the models of AFP (Table 3B), only the CRP level and Glasgow index were significantly associated with the AFP (p<0.05). We also found that the CRP level and Glasgow index were useful for predicting the absence or presence of PVT, as shown in (Table 3D). However, NLR was significant in the model with Glasgow index, but not in the model with CRP (Table 3D). Furthermore, there were no statistically significant variables for the model of multifocality (Table 3C; p>0.05).

### Receiver operating characteristic (ROC) curves for PLR, NLR and CRP

**Figure 1:**
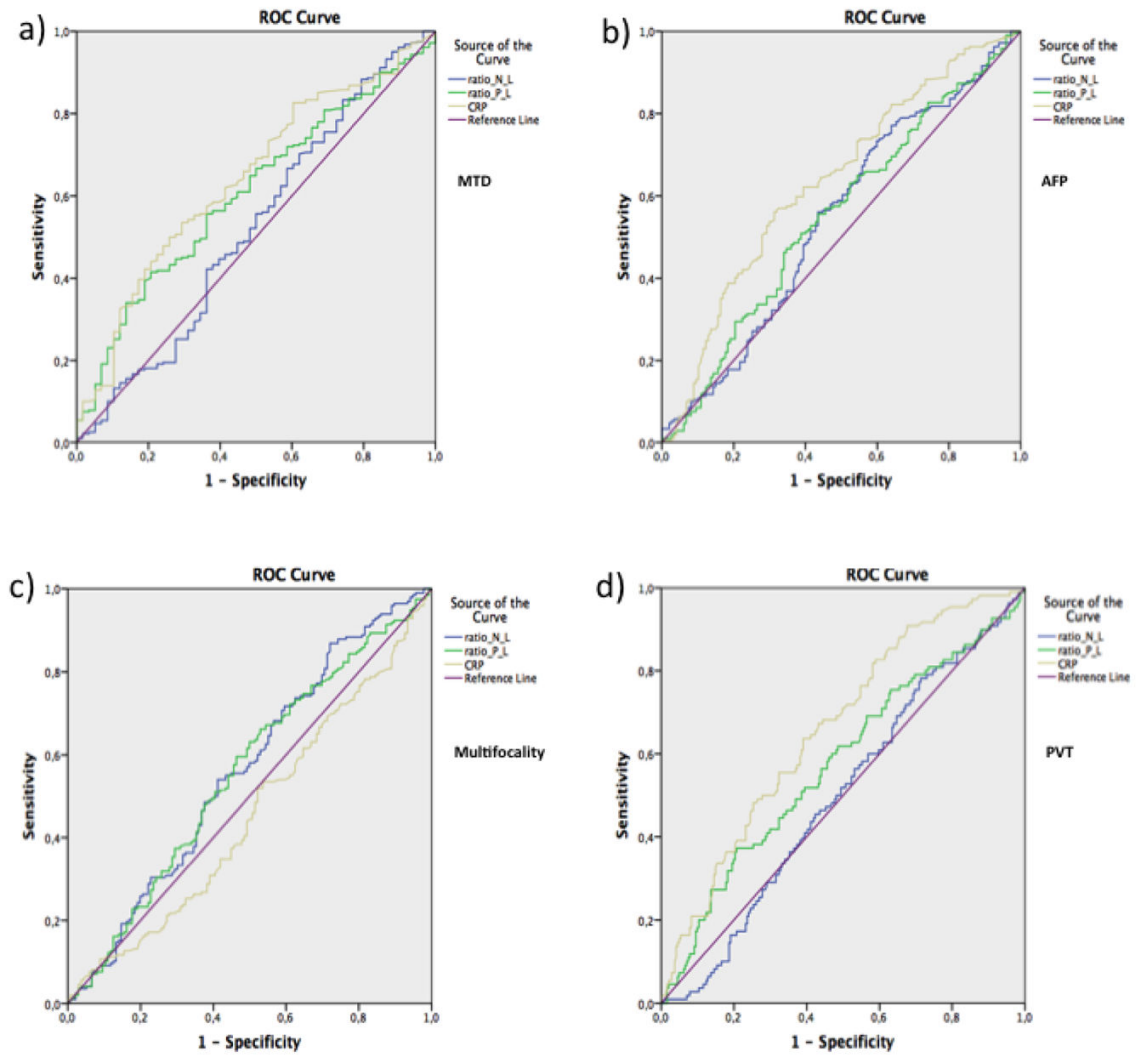
Results of the receiver operation characteristic (ROC) curve of PLR, NLR and CRP for (a) Maximum Tumor Diameter (MTD), (b) Alpha-fetoprotein (AFP), (c) Multifocality, and (d) Portal Vein Thrombosis (PVT) groups

A ROC curve analysis was then performed (Table 4). Our results clearly indicated that AUC for the PLR and CRP was statistically significant for high versus low MTD and presence versus absence of PVT (Table 4). CRP alone was significant for high versus low AFP. Besides, the AUC values of the PLR, NLR and CRP were not statistically significant for multifocality (p>0.05). ROC curves of PLR, NLR and CRP for four parameters are shown in (Figure 1).

## Discussion

In order to better understand their significance, we have examined the associations of 3 commonly used systemic inflammatory markers with each of 4 HCC tumour parameters that reflect HCC aggressiveness. The PLR ratio and the CRP level were each significantly higher in patients with high MTD and positive PVT. Higher CRP levels also significantly associated with higher AFP values. The calculated p-value for the comparison of PLR ratio between multifocal and unifocal patients was near the significance threshold (p<0.05). We thus considered the significance of PLR as a marker to be inconclusive, until more patient data are obtained to accurately elucidate its significance in relation to tumour parameters. The NLR ratio was not found to be a significant marker among the 4 tumour parameters.

When the 4 tumour parameters were included separately in the linear regression model, MTD was the only parameter to predict PLR (Table 2). Our results suggest that patients with MTD≥3cm are almost 3 times more likely to have CRP>10 and nearly twice as likely to have a Glasgow index (CRP plus albumin) = 2. Similarly, patients with AFP≥20 or positive PVT were almost twice as likely to have CRP>10. In the final models of the 4 tumour parameters (Table 3), we found that the PLR and NLR ratios might have potential to predict MTD and PVT, respectively. Although, the PLR and NLR ratios were statistically significant, their corresponding p-values were near the threshold, and our data set might be insufficiently large to be used in making predictions. CRP was a significant variable for MTD, AFP and PVT, while Glasgow index was a significant variable for all the final models-MTD, AFP, PVT and multifocality. Thus, Glasgow index is likely to be a “one-size fits all” predictor for the all 4 tumour parameters. In summary, CRP/Glasgow index has more general use, but for MTD, PLR is far more sensitive a discriminator.

The results of AUC for the PLR ratio and CRP were statistically significant in the MTD and PVT groups. CRP alone was significant for high versus low AFP. In our results (Table 4), CRP had the highest AUC to classify the MTD, AFP and PVT groups.

CRP is a non-specific inflammatory marker that has long been recognized as associated with various inflammatory diseases including coronary artery disease and cancer [1,3,31–33]. Amongst cancers, it has been particularly related to survival amongst gastrointestinal and urothelial cancers, and more recently for HCC [5–10]. CRP is synthesized by hepatocytes, particularly under the control of IL-6, but also of IL-1 and TNF. HCC is particularly associated with inflammation in most cases, due to either chronic hepatitis B, chronic hepatitis C, alcoholism or metabolic syndrome. The development of HCC usually involves several stages, including fibrosis and cirrhosis, both of which are inflammation-associated [34]. The role of the inflammation has been subject to increasing speculation and some experimentation. Inflammation is thought to induce a microenvironment that is involved in DNA damage, tumour growth and angiogenesis. This appears to involve a 2-way process, in which inflammation can be seen as a response to growing tumour cells and also is involved in their growth and invasiveness [33,35]. Various mechanisms appear to be involved, including the presence of tumour growth inducing inflammation and plasma CRP, as well as the tumours directly producing various inflammatory cytokines, including CRP, IL-6 and IL-8, which in turn induce hepatic CRP. Thus, CRP appears to be elevated both locally and systemically. It can thus be seen as a biomarker for the systemic bodily response to growing cancer, but also as a locally-acting mediator of inflammation-associated cancer growth and invasion. CRP has recently been included in a clinically useful prognostication schema (together with serum albumin levels) for GI cancers in general, including HCC, called the Glasgow Index [10–17,19–21]. Other indices for systemic inflammation have recently been reported, especially PLR and NPR [22–28].

In this context, we addressed in this work the possible mechanisms that might underlie the prognostic usefulness of CRP, PLR and NLR. A working hypothesis that we have tested here, is that each or any of CRP, PLR or NLR might be related to indices of tumour aggressiveness, namely MTD, AFP, PVT and multifocality, as an explanation of their prognostic ability [30,36,37]. We found CRP was significantly related to MTD, AFP and PVT, and the Glasgow Index to MTD and AFP. Areas under ROC curves showed that the areas for PLR and CRP were statistically significant for high versus low MTD and for presence versus absence of PVT, and that CRP only was significant for high versus low AFP. However, the function, biological role and significance in determining HCC prognosis is still unclear. In the current study, we report only an association between plasma CRP levels or PLR ratio and indices of HCC aggressiveness.

These analyses suggest that the prognostic usefulness of the inflammatory markers PLR and CRP (but not NLR) may be due to their reflection of parameter values for tumour growth and invasiveness, but do not explain the mechanisms involved, nor do they address whether CRP and PLR are mechanistically involved in these parameters for tumour aggressiveness, or are just reactive reflections of these tumour behaviours.

## Figures and Tables

**Table 1:** Comparisons of the neutrophils-to-lymphocyte ratio (NLR), the platelet-to-lymphocyte ratio (PLR) and the C-reactive protein (CRP) level between HCC patients

Variable	Group	Values	n	Mean±SD	Median (IQR)	Min-Max	p^a^
MTD	<3	NLR	65	4.33±4.33	2.97 (3.82)	0.37–26.50	0.216
	≥3		339	4.58±4.77	3.39 (2.86)	0.77–46.67	
	<3	PLR	63	0.11±0.08	0.09 (0.08)	0.03–0.44	0.002*
	≥3		329	0.17±0.14	0.13 (0.14)	0.02–0.96	
	<3	CRP	59	8.03±15.96	2.08 (6.17)	0.10–81.80	0.001*
	≥3		284	19.19±32.63	5.34 (22.18)	0.10–192.00	
AFP	<20	NLR	163	4.09±3.25	3.03 (2.96)	0.37–18.78	0.101
	≥20		261	4.81±5.39	3.56 (2.90)	0.88–46.67	
	<20	PLR	160	0.15±0.14	0.11 (0.11)	0.02–0.88	0.061
	≥20		253	0.16±0.13	0.13 (0.13)	0.02–0.96	
	<20	CRP	149	14.93±34.39	3.13 (10.20)	0.10–192.00	<0.001*
	≥20		215	20.97±32.41	8.02 (25.64)	0.10–256.00	
Multifocality	Unifocal	NLR	240	4.69±4.79	3.48 (3.10)	0.77–46.67	0.087
	Multifocal		156	4.36±4.66	3.15 (3.03)	0.37–39.29	
	Unifocal	PLR	230	0.16±0.13	0.13 (0.13)	0.02–0.96	0.041*
	Multifocal		154	0.14±0.13	0.10 (0.11)	0.02–0.88	
	Unifocal	CRP	200	16.85±31.59	4.16 (14.95)	0.10–192.00	0.191
	Multifocal		137	18.35±28.91	5.46 (25.45)	0.10–179.00	
PVT	−	NLR	283	4.68±5.03	3.32 (3.05)	0.37–46.67	0.895
	+		135	4.21±3.89	3.47 (2.83)	0.68–33.86	
	−	PLR	275	0.15±0.13	0.11 (0.11)	0.02–0.96	0.034*
	+		131	0.17±0.13	0.14 (0.16)	0.02–0.63	
	−	CRP	244	14.39±28.04	3.32 (13.00)	0.10–180.00	<0.001*
	+		110	28.13±41.83	10.70 (29.98)	0.10–256.00	

SD, Standard Deviation; IQR, Interquartile Range; Min, Minimum; Max, Maximum;

a Wilcoxon rank-sum (Mann-Whitney) test;

* p-value<0.05 is significant

**Table 2:** Regression models on single variables and final models: (A) Linear regression models of the platelet-to-lymphocyte ratio (PLR) on single variables. (B) Logistic regression models of C-reactive protein (CRP) (≤*10/>10*) on single variables. (C) Logistic regression models of Glasgow index (<*2/=2*) on single variables. (D) Logistic regression model of CRP (≤*10/>10*) on final model. (E) Logistic regression model of Glasgow index (<*2/=2*) on final model

	Models on single variables									
	(A)				(B)			(C)		
Parameter	β	se(β)	p	95% C.I.	OR	p	95% C.I.	OR	p	95% C.I.
MTD (<3/≥3)	0.052	0.018	0.004*	0.017 to 0.088	3.144	0.002*	1.529 to 6.463	2.957	0.007*	1.347 to 6.491
AFP (<20/≥20)	0.013	0.013	0.329	−0.013 to 0.039	2.269	<0.001*	1.438 to 3.582	2.404	<0.001*	1.471 to 3.929
Multifocality (Unifocal/Multifocal)	−0.019	0.014	0.173	−0.046 to 0.008	1.436	0.116	0.914 to 2.255	1.878	0.009*	1.168 to 3.019
PVT (−/+)	0.022	0.014	0.111	−0.005 to 0.050	2.211	0.001*	1.393 to 3.507	2.032	0.004*	1.261 to 3.274
	**Final Model**									
					**(D)**			**(E)**		
Parameter^1^					OR	p	95% C.I.	OR	p	95% C.I.
MTD (<3/≥3)					2.860	0.007*	1.328 to 6.160	2.674	0.024*	1.142 to 6.261
AFP (<20/≥20)					1.725	0.030*	1.053 to 2.826	1.765	0.039*	1.030 to 3.026
Multifocality (Unifocal/Multifocal)					-	-	-	1.563	0.084	0.942 to 2.595
PVT (−/+)					1.785	0.024*	1.080 to 2.949	1.620	0.081	0.942 to 2.788

β: coefficient; se(β): standard error of coefficient; OR, Odds-Ratio; C.I.: confidence interval; AFP, Alpha-fetoprotein; MTD, Maximum Tumor Diameter; PVT, Portal Vein Thrombosis.

1 All multiple logistic regression final models were executed on all these variables, included together in the model, and selected with backward stepwise method

* p-value<0.05 is significant

**Table 3:** Final models of logistic regression analysis for (A) Maximum Tumor Diameter (MTD), (B) Alpha-fetoprotein (AFP), (C) Multifocality and (D) Portal Vein Thrombosis (PVT) groups

	(A)			(B)			(C)			(D)		
Parameter^1^	OR	p	95% C.I.	OR	p	95% C.I.	OR	p	95% C.I.	OR	p	95% C.I.
PLR	26.338	0.046*	1.056 to 657.005	-	-	-	-	-	-	-	-	-
NLR	-	-	-	-	-	-	-	-	-	0.947	0.094	0.888 to 1.009
CRP (>10)	2.662	0.009*	1.279 to 5.543	2.329	<0.001*	1.470 to 3.689	1.466	0.098	0.932 to 2.306	2.301	<0.001*	1.441 to 5.674
PLR	33.128	0.034*	1.304 to 841.785	-	-	-	-	-	-	5.083	0.073	0.860 to 30.038
NLR	-	-	-	-	-	-	-	-	-	0.932	0.046*	0.869 to 0.999
Glasgow index (=2)	2.562	0.021*	1.155 to 5.683	2.381	0.001*	1.456 to 3.895	1.875	0.010*	1.165 to 3.017	1.945	0.008*	1.192 to 3.175

OR, Odds-Ratio; C.I.: confidence interval; AFP, Alpha-fetoprotein; MTD, Maximum Tumor Diameter; PVT, Portal Vein Thrombosis.

* p-value<0.05 is significant.

1 All multiple logistic regression final models were executed on all these variables, included together in the model, and selected with backward stepwise method

**Table 4:** Results of the area under the receiver operating characteristic (ROC) curve with 95% confidence intervals of PLR, NLR and CRP for Maximum Tumor Diameter (MTD), Alpha-fetoprotein (AFP), Multifocality and Portal Vein Thrombosis (PVT) groups

	Parameter	Area under curve	Standard error	P value	95% C.I.
MTD (<3/≥3)	PLR	0.607	0.038	0.010*	0.533–0.682
	NLR	0.523	0.044	0.575	0.436–0.610
	CRP	0.642	0.038	0.001*	0.566–0.717
AFP (<20/≥20)	PLR	0.551	0.031	0.102	0.490–0.611
	NLR	0.544	0.031	0.155	0.483–0.606
	CRP	0.635	0.030	<0.001*	0.577–0.694
Multifocality (unifocal/multifocal)	PLR	0.555	0.032	0.088	0.492–0.618
	NLR	0.561	0.033	0.057	0.498–0.625
	CRP	0.456	0.032	0.168	0.393–0.519
PVT (+/−)	PLR	0.575	0.034	0.024*	0.509–0.641
	NLR	0.498	0.032	0.960	0.435–0.562
	CRP	0.660	0.030	<0.001*	0.600–0.719

C.I.: confidence interval.

* p-value<0.05 is significant

## Abbreviations

HCC: hepatocellular carcinoma
PVT: portal vein thrombosis
AFP: alpha-fetoprotein
MTD: maximum tumour diameter
CRP: C-reactive protein
PLR: platelet lymphocyte ratio
NLR: neutrophil lymphocyte ratio
CT: computerized axial tomography
MRI: magnetic resonance imaging

## References

[1] Pepys MB, Baltz ML (1983). Acute phase proteins with special reference to C-reactive protein and related proteins (pentaxins) and serum amyloid A protein. Adv Immunol.

[2] Arcone R, Gualandi G, Ciliberto G (1998). Identification of sequences responsible for acute-phase induction of human C-reactive protein. Nucleic Acids Res.

[3] Toniatti C, Arcone R, Majello B, Ganter U, Arpaia G (1990). Regulation of the human C-reactive protein gene, a major marker of inflammation and cancer. Mol Biol Med.

[4] Ganter U, Arcone R, Toniatti C, Morrone G, Ciliberto G (1989). Dual control of C-reactive protein gene expression by interleukin-1 and interleukin-6. EMBO J.

[5] Fabris C, Pirisi M, Soardo G, Toniutto P, Falleti E (1996). Diagnostic usefulness of acute-phase protein measurement in hepatocellular carcinoma. Cancer Invest.

[6] Hashimoto K, Ikeda Y, Korenaga D, Tanoue K, Hamatake M (2005). The impact of preoperative serum C-reactive protein on the prognosis of patients with hepatocellular carcinoma. Cancer.

[7] Sieghart W, Pinter M, Hucke F, Graziadei I, Schöniger-Hekele M (2013). Single determination of C-reactive protein at the time of diagnosis predicts long-term outcome of patients with hepatocellular carcinoma. Hepatology.

[8] Shin JH, Kim CJ, Jeon EJ, Sung CO, Shin HJ (2015). Overexpression of C-reactive protein as a poor prognostic marker of resectable hepatocellular carcinomas. J Pathol Transl Med.

[9] McMillan DC1, Crozier JE, Canna K, Angerson WJ, McArdle CS (2007). Evaluation of an inflammation-based prognostic score (GPS) in patients undergoing resection for colon and rectal cancer. Int J Colorectal Dis.

[10] Ishizuka M, Kubota K, Kita J, Shimoda M (2012). Impact of an inflammation-based prognostic system on patients undergoing surgery for hepatocellular carcinoma: a retrospective study of 398 Japanese patients. Am J Surg.

[11] Horino K, Beppu T, Kuroki H, Mima K, Okabe H (2013). Glasgow prognostic score as a useful prognostic factor after hepatectomy for hepatocellular carcinoma. Int J Clin Oncol.

[12] Li MX, Bi XY, Li ZY, Huang Z, Han Y (2015). Prognostic role of glasgow prognostic score in patients with hepatocellular carcinoma: a systematic review and meta-analysis. Medicine (Baltimore).

[13] Shiba H, Horiuchi T, Sakamoto T, Furukawa K, Shirai Y (2017). Glasgow prognostic score predicts therapeutic outcome after hepatic resection for hepatocellular carcinoma. Oncol Lett.

[14] Kinoshita A1, Onoda H, Imai N, Iwaku A, Oishi M (2012). Comparison of the prognostic value of inflammation-based prognostic scores in patients with hepatocellular carcinoma. Br J Cancer.

[15] Pinato DJ, Stebbing J, Ishizuka M, Khan SA, Wasan HS (2012). A novel and validated prognostic index in hepatocellular carcinoma: the inflammation based index (IBI). J Hepatol.

[16] Aino H, Sumie S, Niizeki T, Kuromatsu R, Tajiri N (2016). The systemic inflammatory response as a prognostic factor for advanced hepatocellular carcinoma with extrahepatic metastasis. Mol Clin Oncol.

[17] Chan SL, Chan AW, Chan AK, Jian P (2017). Systematic evaluation of circulating inflammatory markers for hepatocellular carcinoma. Liver Int.

[18] Arcone R, Gualandi G, Ciliberto G (1988). Identification of sequences responsible for acute-phase induction of human C-reactive protein. Nucleic Acids Res.

[19] Hu RH, Lee PH, Yu SC (1999). Secretion of acute-phase proteins before and after hepatocellular carcinoma resection. J Formos Med Assoc.

[20] Zheng J, Seier K, Gonen M, Balachandran VP, Kingham TP (2017). Utility of Serum Inflammatory Markers for Predicting Microvascular Invasion and Survival for Patients with Hepatocellular Carcinoma. Ann Surg Oncol.

[21] He CB, Lin XJ (2017). Inflammation scores predict the survival of patients with hepatocellular carcinoma who were treated with transarterial chemoembolization and recombinant human type-5 adenovirus H101. PLoS One.

[22] Zhao Y, Si G, Zhu F, Hui J, Cai S (2017). Prognostic role of platelet to lymphocyte ratio in hepatocellular carcinoma: a systematic review and meta-analysis. Oncotarget.

[23] Xue TC, Jia QA, Ge NL, Zhang BH, Wang YH (2015). The platelet-to-lymphocyte ratio predicts poor survival in patients with huge hepatocellular carcinoma that received transarterial chemoembolization. Tumour Biol.

[24] Li X, Chen ZH, Xing YF, Wang TT, Wu DH (2015). Platelet-to-lymphocyte ratio acts as a prognostic factor for patients with advanced hepatocellular carcinoma. Tumour Biol.

[25] Yang HJ, Jiang JH, Liu QA, Zhou CM, Du YF (2017). Preoperative platelet-to-lymphocyte ratio is a valuable prognostic biomarker in patients with hepatocellular carcinoma undergoing curative liver resection. Tumour Biol.

[26] Tian XC, Liu XL, Zeng FR, Chen Z, Wu DH (2016). Platelet-to-lymphocyte ratio acts as an independent risk factor for patients with hepatitis B virus-related hepatocellular carcinoma who received transarterial chemoembolization. Eur Rev Med Pharmacol Sci.

[27] Ma W, Zhang P, Qi J, Gu L, Zang M (2016). Prognostic value of platelet to lymphocyte ratio in hepatocellular carcinoma: a meta-analysis. Sci Rep.

[28] Chen K, Zhan MX, Hu BS, Li Y (2018). Combination of the neutrophil to lymphocyte ratio and the platelet to lymphocyte ratio as a useful predictor for recurrence following radiofrequency ablation of hepatocellular carcinoma. Oncol Lett.

[29] Ni XC, Yi Y, Fu YP, He HW, Cai XY (2015). Prognostic value of the modified glasgow prognostic score in patients undergoing radical surgery for hepatocellular carcinoma. Medicine (Baltimore).

[30] Akkiz H, Carr B, Yalcin KK, Guerra V, Kuran S (2018). Characteristics of hepatocellular carcinoma aggressiveness factors in Turkish patients. Oncology.

[31] Drahovsky D, Dunzendorfer U, Ziegenhagen G, Drahovsky M, Kellen JA (1981). Reevaluation of C-reactive protein in cancer sera by radioimmunoassay and radial immunodiffusion. I. Diagnostic value and use in battery of conventional tumor markers. Oncology.

[32] Mahmoud FA, Rivera NI (2002). The role of C-reactive protein as a prognostic indicator in advanced cancer. Curr Oncol Rep.

[33] Shrotiya S, Walsh D, Bennani-Baiti N, Thomas S, Lorton C (2015). C-Reactive Protein is an important biomarker for prognosis tumor recurrence and treatment response in adult solid tumors: a systematic review. PLoS one.

[34] Fuxe J, Karlsson MC (2012). TGF beta-induced epithelial mesenchymal transition: a link between cancer and inflammation. Sem in Cancer Biology.

[35] Balkwill F, Mantovani A (2001). Inflammation and cancer: back to Virchow?. Lancet.

[36] Carr BI, Guerra V (2016). A Hepatocellular Carcinoma Aggressiveness Index and Its Relationship to Liver Enzyme Levels. Oncology.

[37] Carr BI, Guerra V, Giannini EG, Farinati F, Ciccarese F (2016). A Liver Index and its Relationship to Indices of HCC Aggressiveness. J Integr Oncol.

